# Ovarian somatic Piwi regulates nurse cell proliferation and oocyte specification in *Drosophila*

**DOI:** 10.17912/micropub.biology.000324

**Published:** 2020-11-18

**Authors:** Lauren E Gonzalez, Gina Zhu, Haifan Lin

**Affiliations:** 1 Yale Stem Cell Center, Yale School of Medicine, New Haven, CT 06519, USA; 2 Department of Genetics, Yale School of Medicine, New Haven, CT 06519, USA; 3 Yale College, New Haven, CT 06511, USA; 4 Department of Cell Biology, Yale School of Medicine, New Haven, CT 06519, USA

**Figure 1. Knockdown of piwi expression in all ovarian somatic cells by tj-GAL4 results in abnormal ovarian morphology reflective of GSC proliferation and differentiation defects:**
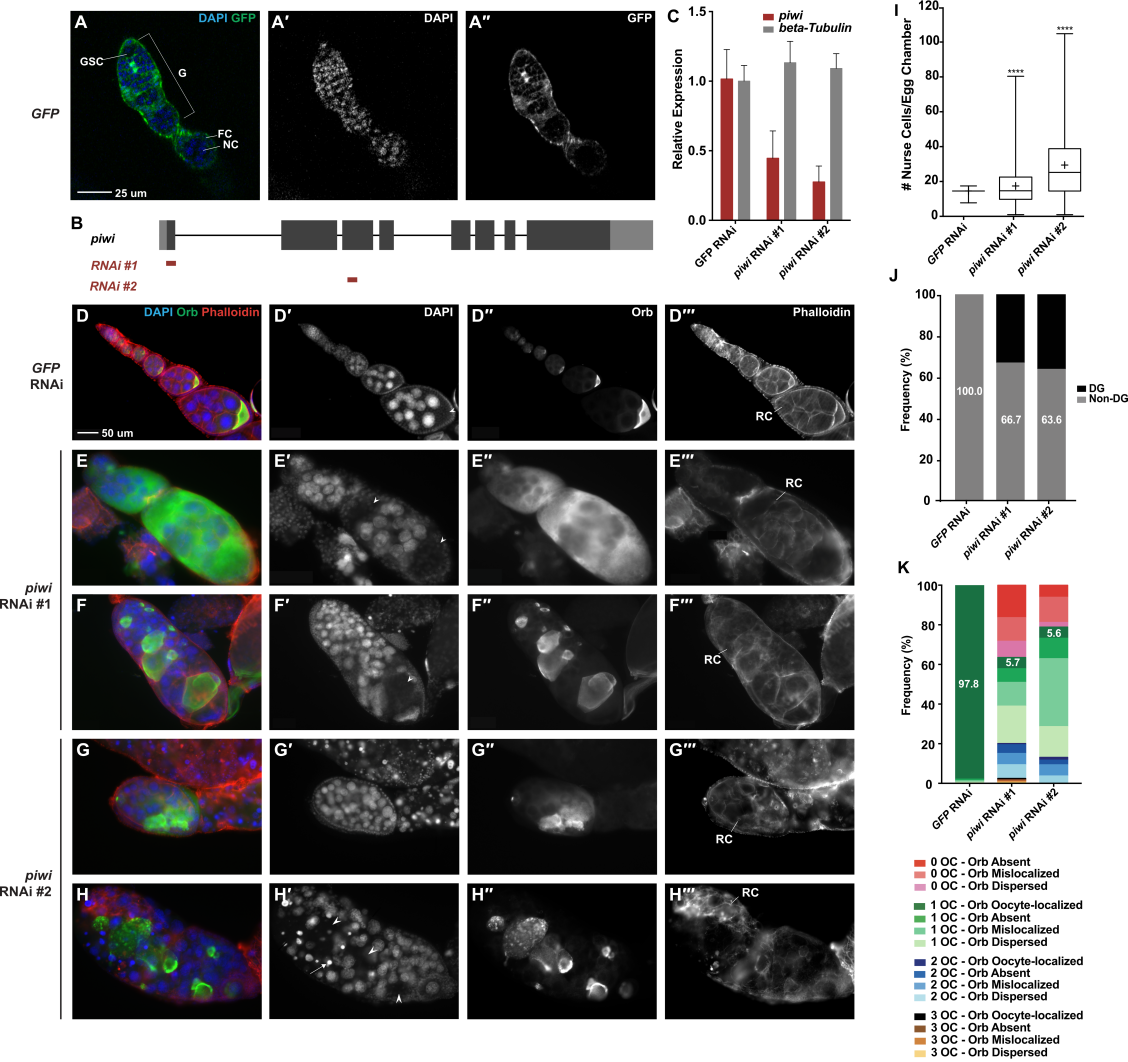
**(A – A′′)** *tj-GAL4* driving *UASp-GFP* demonstrates that the driver is expressed in all ovarian somatic cells. **(B)** A schematic drawing of *piwi*gene structure and target regions of *piwi* RNAi #1 and *piwi* RNAi #2. **(C)** RT-qPCR demonstrates that *tj-GAL4* driving *piwi* RNAi #1 or *piwi* RNAi #2 reduces *piwi* expression in all ovarian somatic cells compared to the control. **(D – H′′′)** *tj-GAL4* RNAi experiments show that *piwi* depletion in ovarian somatic cells causes dramatic morphological defects.DAPI staining marks the nuclei of nurse cells and presumptive oocyte nuclei, Orb is the oocyte-specific protein, and Phalloidin marks F-actin structures, including ring canals. Presumptive oocytes are indicated with arrowheads. Degenerate nurse cells, identified by pyknotic nuclei, are indicated with arrows. **(D – D′′′)** *tj-GAL4* driving *GFP* RNAi demonstrates the normal egg chamber morphology of 15 nurse cells, 1 presumptive oocyte, and oocyte-localized Orb staining. **(E – E′′′)** 15 nurse cells are observed, but two presumptive oocytes are identified. The Orb protein localization pattern is dispersed throughout the egg chamber. **(F – F′′′)** A tumorous number of nurse cells are observed and one presumptive oocyte is observed. However, the Orb protein is mislocalized to non-oocyte germline cells. **(G – G′′′)** A tumorous number of nurse cells is observed and no presumptive oocytes are observed; however, Orb staining is still observed and is mislocalized. **(H – H′′′)** Degenerate nurse cells are observed, identifiable by their bright pyknotic nuclei. No presumptive oocyte is observed, but Orb staining is still present and thus considered mislocalized. **(I)** A box and whisker plot of the number of nurse cells per egg chamber for each genotype. **** represents a p-value < 0.0001 compared to *tj-GAL4>GFP* RNAi, unpaired t-test. **(J)** The frequency of egg chambers that had non-degenerate and degenerate nurse cells. **(K)** The frequency of egg chambers with zero, one, two, or three presumptive oocytes (OC) and with oocyte-localized, absent, mislocalized, or dispersed Orb staining. The number of egg chambers counted for each genotype is as follows: *tj-GAL4*>*GFP* RNAi = 1210; *tj-GAL4*>*piwi* RNAi #1 = 159; *tj-GAL4*> *piwi* RNAi #2 = 213. DG=degenerate, F= follicle cell, G=germarium, GSC=germline stem cell, NC=nurse cell, OC=oocyte, RC=ring canal.

## Description

The development of germ cells requires communication between the germline and the soma, a process that has been extensively characterized within the *Drosophila* ovary. Throughout the lifetime of the female fly, germline stem cells (GSCs) continually self-renew and produce differentiating daughter cells (cystoblasts), and this process relies on communication with the ovarian somatic cells (OSCs) within the stem cell niche: terminal filament cells, cap cells, and escort cells (Lin and Spradling 1993, Xie and Spradling 2000). Cystoblasts then go through four rounds of cell division with incomplete cytokinesis to produce a germline cyst of precisely 16 sister cells (cystocytes) with their cytoplasm connected by intercellular cytoplasmic bridges called ring canals. One of the 16 cystocytes subsequently becomes the oocyte, while the other 15 become nurse cells. Nurse cells endoreplicate to become extremely polyploid, and produce and transport mRNAs and proteins into the oocyte via ring canals to promote its maturation. Due to this unidirectional flow, the oocyte can be easily identified even within the earliest stage egg chambers by the localization of proteins such as Orb (Christerson and McKearin 1994, Lantz *et al.* 1994), and at later stages it is easily recognizable from its very small nucleus-to-cytoplasm ratio. This 16-cell germline cyst is encapsulated by a monolayer of somatic follicle cells, which support oocyte development throughout the remainder of oogenesis.

Early in the characterization of the stem cell niche, genetic screens revealed that Piwi, the founding member of the evolutionarily conserved PIWI/AGO protein family which is now well-known for its transposon suppression function, is required for the maintenance of GSC stem-ness (Cox *et al.* 1998, Cox *et al.* 2000). The ovaries of null *piwi* mutants lack germline cells almost entirely, reflecting a role for *piwi* in GSC self-renewal. Mosaic analysis showed that *piwi* mutations in germline cells of the ovary did not impair GSC self-renewal (Cox *et al.* 1998), suggesting that *piwi* expression in the ovarian soma is required for GSC self-renewal, and thereby implicating Piwi in soma-to-germline crosstalk.

The vast majority of further studies into the functions of *piwi* and its associated small noncoding PIWI-interacting RNA (piRNA) have focused on germline cells. It has been discovered that Piwi largely localizes to the nucleus, where it is guided by piRNA to target nascent RNA. There, Piwi impacts gene expression by modulating chromatin modifications (Brower-Toland *et al.* 2007, Wang and Elgin 2011, Sienskiet *et al.* 2012). Piwi and other PIWI proteins can also regulate gene expression post-transcriptionally to influence a variety of developmental processes (Robine *et al.* 2009, Saito *et al.* 2009). In addition to the many studies into the role of Piwi within the *Drosophila* germline, some studies have investigated its ovarian somatic function. Two such studies revealed that *piwi* expression in escort cells prevents the formation of GSC tumors (Jin *et al.* 2013, Ma *et al.* 2014), indicating that somatic Piwi can influence the differentiation of germline cells.

To further investigate the function of Piwi in OSCs in regulating germline cells, we used *traffic jam GAL4* (*tj-GAL4)*, which expresses in all somatic cells but not germline cells of the ovary (Fig 1 A- A′′, Olivieri *et al.* 2010), to deplete *piwi* expression in all OSCs (herein abbreviated as *piwi*-sKD). The two anti-*piwi* RNAi lines we used, “*piwi* RNAi #1*”* and “*piwi* RNAi #2*,*” target different regions of the *piwi* mRNA (Figure 1B) and result in different degrees of *piwi* knockdown (Figure 1C), providing an opportunity to explore Piwi’s ovarian somatic function in an expression-level-dependent way. We will refer to the *piwi* somatic knockdown as *piwi*-sKD #1 and *piwi-*sKD #2.

*Piwi*-sKDresulted in a variety of defects, including an abnormal number of nurse cells, an increase in the number of degenerate nurse cells, failure of proper oocyte specification, failure of proper Orb localization, loss of polarity in the egg chamber, and a general disorganization of ovarioles (*cf.* Fig. 1D and 1E – H′′′). We observed these defects in very early as well as late stage egg chambers, but limited the quantification of these defects to stage 6-10 egg chambers, because this is when the oocyte becomes morphologically recognizable (a “presumptive oocyte”).

Defects in nurse cell number ranged dramatically: in *piwi*-sKD ovaries, we observed egg chambers with as few as one nurse cell and others with over 100 nurse cells, with the average number significantly above the typical 15 nurse cells in a wild-type egg chamber (Fig 1I). Strikingly, over 30% of *piwi*-sKD egg chambers were “tumorous,” containing more than 30 nurse cells, some of which appeared to be diploid (Fig 1I). We also observed an increased frequency of egg chambers containing degenerate (DG) nurse cells in *piwi-*sKD egg chambers compared to controls (Fig 1J). These nurse cell number abnormalities suggest a role for somatic Piwi in regulating germline cystoblast and/or cystocyte proliferation.

In addition, many *piwi*-sKD egg chambers were abnormal in number and/or specification of oocytes. The number of presumptive oocytes per egg chamber ranged from zero to three. Among these presumptive oocytes, some had correct Orb protein localization (“oocyte-localized”), but other egg chambers lacked any Orb staining (“absent”), contained Orb protein localized to non-oocyte cells (“mislocalized”), or contained Orb protein dispersed throughout the whole egg chamber (“dispersed”). Only 5.7% and 5.6% of *piwi*-sKD #1 and *piwi-*sKD #2 egg chambers, respectively, had one oocyte and oocyte-localized Orb (Fig 1K), indicating that somatic Piwi plays a role in oocyte specification.

The defects in nurse cell number and oocyte specification upon somatic *piwi* depletion reveal novel roles for Piwi in OSCs in regulating the proliferation of germline cells and the specification of the oocyte. This function is distinct from the well-described role of somatic Piwi in GSC maintenance, wherein the loss of Piwi function results in a loss of germline cells altogether. Instead, this *piwi*-sKD phenotype echoes the germline over-proliferation phenotypes observed upon depleting *piwi* expression in testicular somatic cells (Gonzalez *et al.* 2015) or ovarian escort cells (Jin *et al.* 2013, Ma *et al.* 2014).

It is possible that some of the defects we have observed upon *piwi*-sKD are the result of GSC tumors observed by other groups upon *piwi* depletion in escort cells. Typically, a GSC, marked by a cytoskeletal structure called the spectrosome, produces 16-cell cysts connected via the fusome, a branched inter-cellular derivative structure of the spectrosome that traverses ring canals within an egg chamber. The GSC tumor-filled germaria formed by *piwi* depletion in escort cells were described as containing mostly undifferentiated germ cells marked with spectrosomes (Jin *et al.* 2013, Ma *et al.* 2014). The tumorous egg chambers we observed upon *piwi-*sKD may result from the encapsulation of these GSC tumors by follicle cells. However, in the majority of egg chambers, we observe polyploid germ cells that are connected by ring canals, so some cystoblasts must have undergone endoreplication and cell division with incomplete cytokinesis. The fact that the *piwi*-sKD egg chambers often contain many more than 15 nurse cells suggests over-proliferation of cystocytes within a cyst or incorporation of multiple cysts into the same egg chamber, or both.

The possibility that at least some of the aberrant *piwi-*sKD egg chambers contain multiple germline cysts is supported by our observation of many egg chambers with multiple oocytes. However, this explanation likely does not extend to egg chambers containing no presumptive oocyte or dislocalized Orb in *piwi-*sKD egg chambers. In those egg chambers, oocytes are not correctly positioned within the egg chamber, and either oocytes are not properly specified by signaling from follicle cells, or the transportation of oocyte-specific markers, like Orb, into the oocyte does not occur.

Overall, our study reveals the function of *piwi* in somatic cells of the ovary to regulate germline cyst formation and oocyte specification. More cell-type specific *piwi* depletion will further reveal whether Piwi functions in particular sub-types of ovarian somatic cells to regulate the above soma-to-germline signaling during oogenesis.

## Methods

***Drosophila husbandry and genetics***

All *Drosophila* stocks were raised on standard agar/molasses medium and raised at 25°C for experiments. *Traffic jam-GAL4* (*tj-GAL4*) from Kyoto Stock Center (DGRC #104055) was used to express UASp constructs in all somatic cells of the ovary. *UASp-GFP* (BDSC #1521) was used to verify the expression pattern of *tj-GAL4*. Two anti-Piwi RNAi lines (“*piwi* RNAi #1” and “*piwi* RNAi #2*”* in this study) were used to knock down *piwi* expression. *piwi* RNAi #1 targets exon 2 of the *piwi* mRNA and is BDSC stock #37483; *piwi* RNAi #2 targets exon 3 of the *piwi* mRNA and was a gift from T. Xie, Stowers Institute For Medical Research, Kansas City, MO. *GFP* RNAi (BDSC stock #41550) was used as a negative control. To generate *GAL4/UASp* flies for analysis, two males carrying the *GAL4* driver were crossed with three virgin females carrying the *UASp* construct. *GAL4/UASp* females were aged for two to three days in a ratio of 2:1 with *w^1118^* males prior to ovary dissection.

***RNA extraction and RT-qPCR***

Ovaries were dissected from 2-3 day-old females in 1XPBS, then transferred to TRIzol (Thermo Fischer Scientific, #15596018) and RNA was extracted following manufacturer’s instructions. 6-8 pairs of ovaries were grouped together for each sample. 2 µg of total RNA was used for reverse transcription using MultiScribe (Thermo Fischer Scientific, #4311235) following manufacturer’s instructions, and diluted 5x before quantitative PCR using iTaq SYBR MasterMix (BioRad, #172-5125). Relative fold changes were calculated using the ΔΔC_T_ method, with *actin5C* as the reference (F: GAAGAAGTTGCTGCTCTGGTTG; R: GAGCATCGTCTCCGGCAAATC), to determine expression levels of *piwi* (F: TTGTCCAATCATGCTAACCTTCTGGGAT, R: CTTATTTCGATCTTAGCTCGGGGATC) and *beta-Tubulin* (F: TGTCGCGTGTGAAACACTTC, R: AGCAGGCGTTTCCAATCTG).

***Immunostaining***

Ovaries from 2-3 day old females were dissected in 1X PBS and fixed with 200 μL of the following fixing solution (v/v%): PBS (89.5%), 10% Nonidet P-40 (5%), 37% formaldehyde (5.5%). The fixed ovaries were then washed 3 times for 15 minutes each in PBST (PBS and 0.2% Triton X-100). Ovaries were then blocked overnight in 5% NGS at 4^o^C, followed by incubation overnight at 4^o^C in primary antibody diluted in PBST + 2% NGS. Samples were washed three times in PBST and incubated in secondary antibodies overnight at 4^o^C. Samples were washed three times with PBST, stained with DAPI (1:500) and Phalloidin (1:200, ThermoFisher, #R415) for 15 minutes, and mounted in Vectashield mounting media (Vector Labs, #H1000).

The following primary antibodies were used: mouse anti-Piwi 4K5 (1:15,000, made in Lin Lab) and mouse anti-Orb 4H8 (1:300, DSHB). The following conjugated secondary antibodies were used, all at 1:500 dilutions: The Alexa 488-conjugated goat anti-mouse antibody and the Alexa 555-conjugated goat anti-mouse. Orb, Phalloidin, and DAPI were used to qualitatively and quantitatively characterize the observed phenotypes of the egg chambers from all crosses.

***Microscopy and phenotypic characterization***

To observe the GAL4 expression pattern, confocal images of DAPI stained *tj-GAL4>UASp-GFP* ovaries were taken using Leica TCS SP5 Confocal Laser Scanning Microscope. Piwi knockdown samples were analyzed on the ZEISS Axio Imager2 for quantitative and qualitative characterizations.

DAPI and Phalloidin staining was used to identify nurse cells and oocytes. To more accurately quantify the effect of *piwi*-sKD on the proliferation and development of nurse cells, we created four categories to describe how many nurse cells were in an egg chamber: 15 normal nurse cells; 0-14 normal nurse cells; 16-29 normal nurse cells; and ≥30 normal nurse cells, which we referred to as a tumorous number. Degenerate nurse cells were identified by the presence of bright pycnotic nuclei. Any large area of cytoplasm without a polyploid nucleus was considered a presumptive oocyte. We quantified how many egg chambers had zero, one, two, or three presumptive oocytes. We also characterized the localization of the Orb staining based on its relative position with the presumptive oocyte. We created four categories of Orb’s localization pattern: absent, oocyte-localized, mislocalized, and dispersed. If the egg chamber had no trace of Orb staining, the pattern was considered absent. If the Orb staining localized with the presumptive oocyte(s), it was designated oocyte-localized; but if the Orb staining was not localized to the presumptive oocyte and formed discrete patches, it was considered mislocalized. If the Orb staining filled the entire egg chamber, its pattern was considered dispersed.

***Statistical Analysis***

Statistical significance for nurse cell number in *piwi-*sKD was assessed via an unpaired t-test using *tj-GAL4* driving *GFP siRNA* as a control.
